# sendigR: an R package to leverage the value of CDISC SEND datasets for cross-study analysis

**DOI:** 10.3389/ftox.2024.1392686

**Published:** 2024-07-15

**Authors:** K. Snyder, C. M. Sabbir Ahmed, Md Yousuf Ali, S. Butler, Michael DeNieu, W. Houser, B. Paisley, M. Rosentreter, W. Wang, B. Larsen

**Affiliations:** ^1^ US Food and Drug Administration, Silver Spring, MD, United States; ^2^ Oak Ridge Institute for Science and Education, Oak Ridge, TN, United States; ^3^ Labcorp, Madison, WI, United States; ^4^ Bristol Myers Squibb, New Brunswick, NJ, United States; ^5^ Eli Lilly & Co, Indianapolis, IN, United States; ^6^ Bayer AG, Wuppertal, Germany; ^7^ Novo Nordisk, Copenhagen, Denmark

**Keywords:** CDISC Standard for Exchange of Nonclinical Data, toxicology data, cross-study analysis, BioCelerate, United States FDA, sendigR

## Abstract

The CDISC Standard for Exchange of Nonclinical Data (SEND) data standard has created new opportunities for collaborative development of open-source software solutions to facilitate cross-study analyses of toxicology study data. A public–private partnership between BioCelerate and the FDA/Center for Drug Evaluation and Research (CDER) was established in part to develop and publicize novel methods to facilitate cross-study analysis of SEND datasets. As part of this work in collaboration with the Pharmaceutical Users Software Exchange (PHUSE), an R package sendigR has been developed to enable users to construct a relational database from a collection of SEND datasets and then query that database to perform cross-study analyses. The sendigR package also includes an integrated Python package, xptcleaner, which can be used to harmonize the terminology used in SEND datasets by mapping to CDISC controlled terminologies. The sendigR R package is freely available on the comprehensive R Archive Network (CRAN) and at https://github.com/phuse-org/sendigR. An R Shiny web application was included in the R package to enable toxicologists with no coding experience to perform historical control analyses. Experienced R programmers will be able to integrate the package functions into their own custom scripts/packages and potentially contribute improvements to the functionality of sendigR.

sendigR reference manual: https://phuse-org.github.io/sendigR/.

sendigR R Shiny demo app: https://phuse-org.shinyapps.io/sendigR/.

## Introduction

The Standard for Exchange of Nonclinical Data (SEND) provides nonclinical toxicology study data in a structured, standardized electronic format and assists with the exchange of nonclinical study data within and between contract research organizations (CROs) and sponsor companies (SENDIG v3.0, 2011). SEND has been required for submissions to the US FDA Center for Drug Evaluation and Research (CDER) and the Center for Biologics Evaluation and Research (CBER) since December 2016 and March 2023, respectively. Currently, SEND supports a variety of study types: single/repeat-dose general toxicity, carcinogenicity, respiratory and cardiovascular safety pharmacology, and embryofetal reproductive toxicology. With the evolution of SEND and the introduction of new standards (e.g., Developmental and Reproductive Toxicology [DART] and Genetic Toxicity), this list will grow (SENDIG-DART v1.1, 2017; SENDIG-Genetox v1.0, 2023). The US FDA CDER repository having more than 10,000 sponsor-submitted SEND datasets provides the opportunity for large-scale, cross-data analysis of toxicology data, which can ultimately provide safer compounds to patients faster.

**FIGURE 1:**
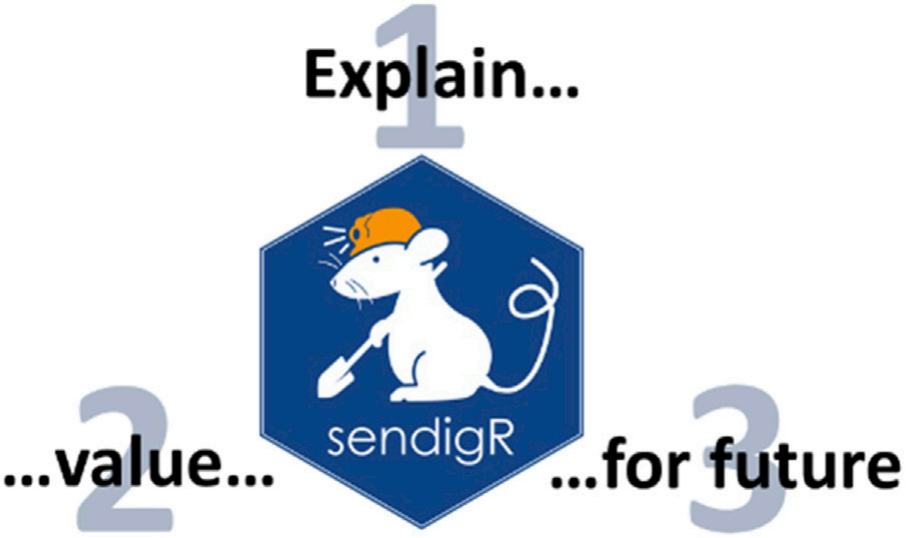
The goals of the sendigR project are to (1) explain and exemplify how to use the sendigR package for harmonizing SEND Transport files and cross-study analysis of historical background control data, (2) demonstrate the value of the sendigR analytic approaches, and (3) provide a foundation for future work/publications.

As there is substantial value in developing tools to facilitate cross-study analysis of SEND data, the BioCelerate consortium has led efforts since 2018 in partnership with the US FDA to identify solutions to enable analytic approaches for SEND datasets (Carfagna et al., 2020). Inconsistent application of the available standards in toxicology data prevents analysis of data across studies, accurate statistical calculations, and sharing of data across organizations. Solutions were needed to ensure that synonymous terms were mapped to a single SEND controlled terminology term for consistency in data (Briggs, 2017; Sato et al., 2018; Sato et al., 2021). Additionally, the team identified gaps in the ability to compare data across studies due to differences in measurements in species, tissues, endpoints, study duration, age, etc. Therefore, normalization methods were needed to enable comparison of disparate endpoints and to account for these variations (Carfagna et al., 2024). Finally, SEND control data can provide insights into background finding incidence for cohorts of animals; however, access to harmonized SEND data for this purpose may be restrictive for individuals without a programming background (Carfagna et al., 2021). Therefore, there was a need to develop an R Shiny user interface to provide an intuitive front-end for non-programmers to explore their SEND control data (Chang et al., 2020; R Core Team, 2022). A large collection of R packages has been collaboratively developed and published on the comprehensive R Archive Network (CRAN) by the pharmaceutical industry for the purposes of ingesting, analyzing, and visualizing CDISC-formatted clinical trial data (Clark et al., 2023; Miller et al., 2024; Pharmaverse, 2024; Straub et al., 2024); however, sendigR is the first R package that facilitates the analysis of CDISC SEND-formatted toxicology study data to be published to the CRAN. In this paper, we discuss the development of these open-source tools and use cases to illustrate their value in the field of toxicology. This paper presents the sendigR package algorithms developed by BioCelerate in collaboration with the FDA and the Pharmaceutical Users Software Exchange (PHUSE) for the purposes given in Figure 1.

## Materials and methods

The sendigR project was designed to facilitate efficient extraction of relevant historical control data from SEND datasets for analysis. An R package (available at https://github.com/phuse-org/sendigR) and user-friendly R Shiny web application (available at https://phuse-org.shinyapps.io/sendigR/) were created to enable querying a database of SEND-formatted toxicology study datasets to retrieve historical control distributions for study endpoints. Shiny (Chang et al., 2020) is an R package, which facilitates deployment of web applications using the R programming language. The R Shiny web application provides a user-friendly interface, making the functionality of the sendigR R package accessible to toxicologist end users who may not be experienced R programmers. The sendigR R package includes a function called sendDashboard, which will launch the web application, enabling users to search and extract historical control data from a SEND database. With the sidebar menu in the app, the user can filter date range, study design, route of administration, species, strain, and sex to obtain the appropriate control animals from the database (Supplementary Figure S1). The main dashboard displays data associated with the selected control animals in several tabs. Upon performing a query, the ANIMALS tab displays metadata on each of the control animals (Supplementary Figure S2). There are three additional tabs in the main dashboard, where each displays data on the selected control animals that are encoded in a corresponding SEND domain, i.e., MI (microscopic findings), LB (laboratory test results), and BW (body weight). Within each domain-specific tab, data can be viewed for individual animals or as aggregated calculations of reference ranges or background incidence rates of findings via another set of tabs (Supplementary Figure S3). Under the Individual Records tab, the raw domain specific data for all control animals can be explored. Under the Aggregate Table tab, for MI, the background incidence rate of histopathology findings within each organ/tissue is displayed (Supplementary Figure S4), whereas for the LB reference, ranges are displayed in the form of mean, standard deviation, and number of observations for each laboratory test result (Supplementary Figure S5). All tables can be sorted in increasing or decreasing order, and rows from tables can be filtered out with specific values provided in columns. Additionally, all tables can be exported as CSV or RDS files for further analysis.

**FIGURE 2:**
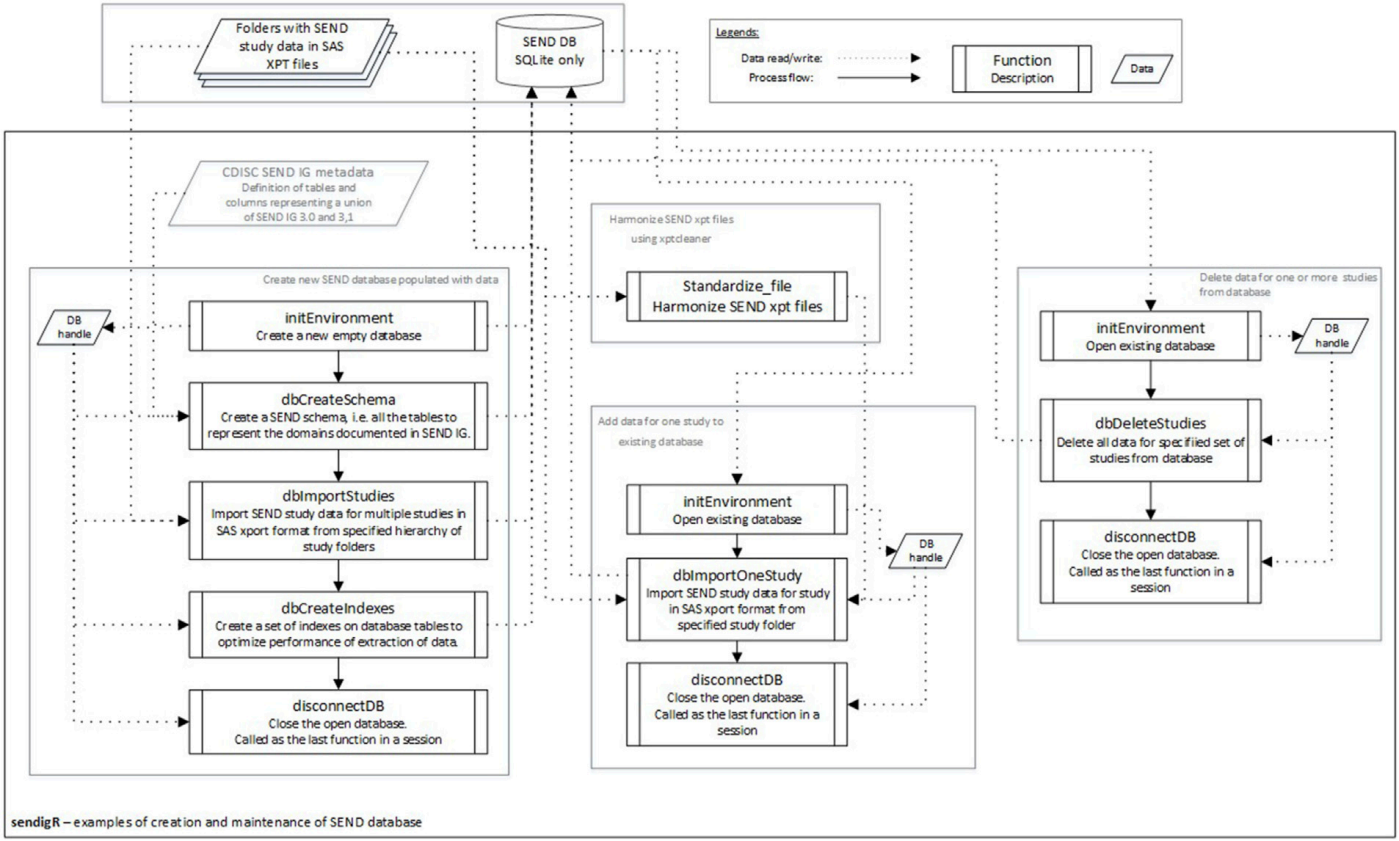
The flow diagram depicts the sequence of function calls to create a new database of SEND data from a collection of studies and how to add or remove individual studies.

SEND datasets consist of several data tables, referred to as domains, which are stored in separate files using the SAS Transport File Format (XPT). The sendigR R package can be used to build and maintain an SQLite database of SEND datasets containing a table for each domain (Figure 2). No transformations are performed when loading XPT files into the database. The initEnvironment function is used to connect to an existing database or create a new empty database. If an empty database is created, the dbCreateSchema function is needed to create tables in the database for each domain in the SEND schema. After creating the SEND schema, multiple studies can be added to the database from a hierarchy study folder using the dbImportStudies, or one study can be added using the dbImportOneStudy function. The dbCreateIndex function can be run to index the database, optimizing its performance. One or multiple studies can be deleted from the existing database using the dbDeleteStudies function. At the end of a session, the disconnectDB function should be used to disconnect from the database.

**FIGURE 3:**
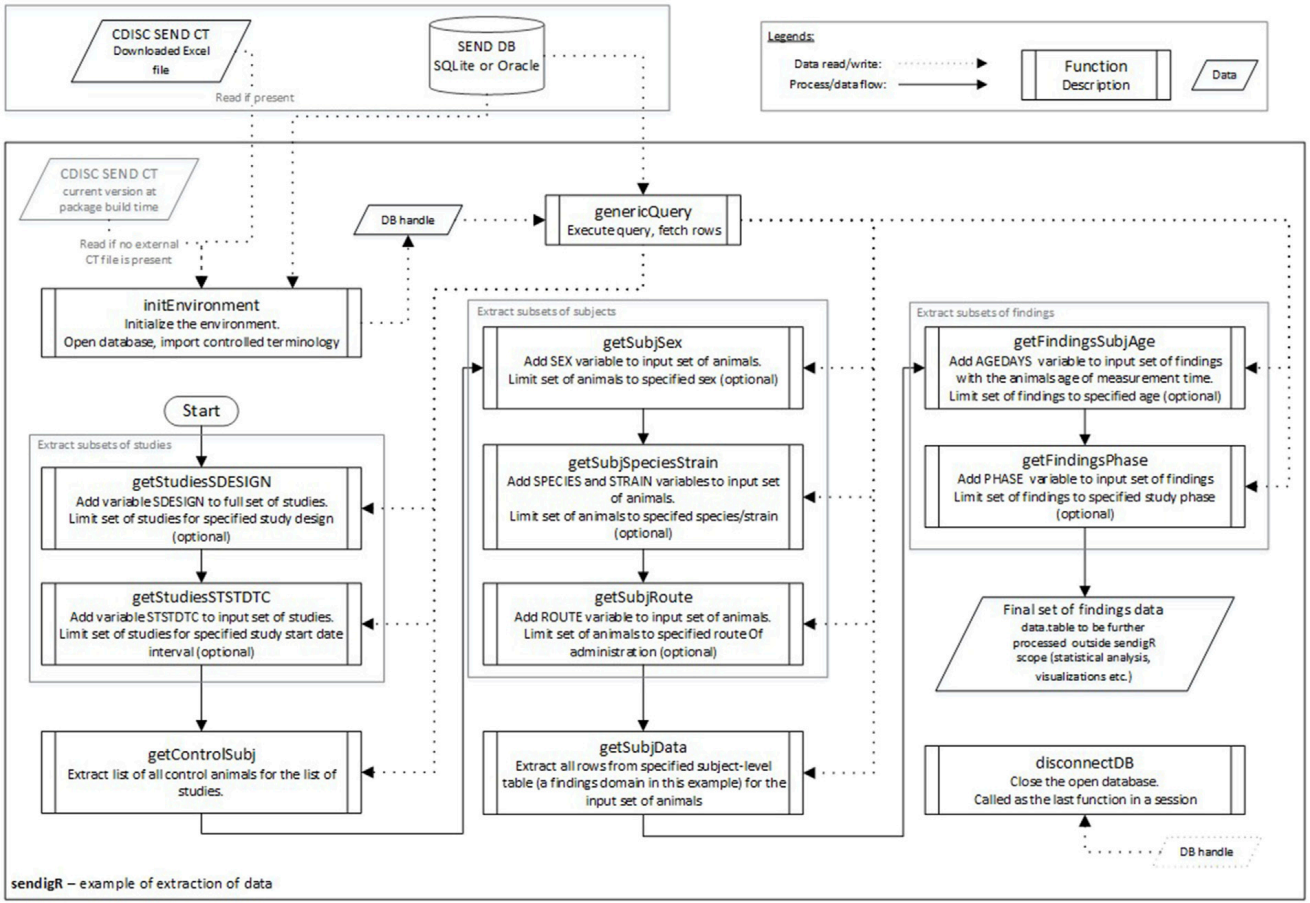
The flow diagram depicts how the various sendigR functions can be called to query the database to extract historical control data.

Figure 3 shows an example of the flow of function calls that would be used to extract relevant historical control data from the database. The initEnvironment function must be called first to connect to an existing database to query. Once the connection is established, a set of functions are used to query the database. These functions can be categorized into two groups: study-level filter functions, i.e., getStudiesSDESIGN and getStudiesSTSTDTC, and subject-level filter functions, i.e., getControlSubj, getSubjSex, getSubjSpeciesStrain, and getSubjRoute. After the selection of control animals has been appropriately filtered, the getSubjData function is called to extract toxicology study results for these animals associated with a particular SEND domain, e.g., MI (microscopic findings). Finally, getFindingsSubjAge and getFinidngsPhase can be called to facilitate filtering of study result data based on the age of the animal and phase of the study during which they were observed.

**FIGURE 4:**
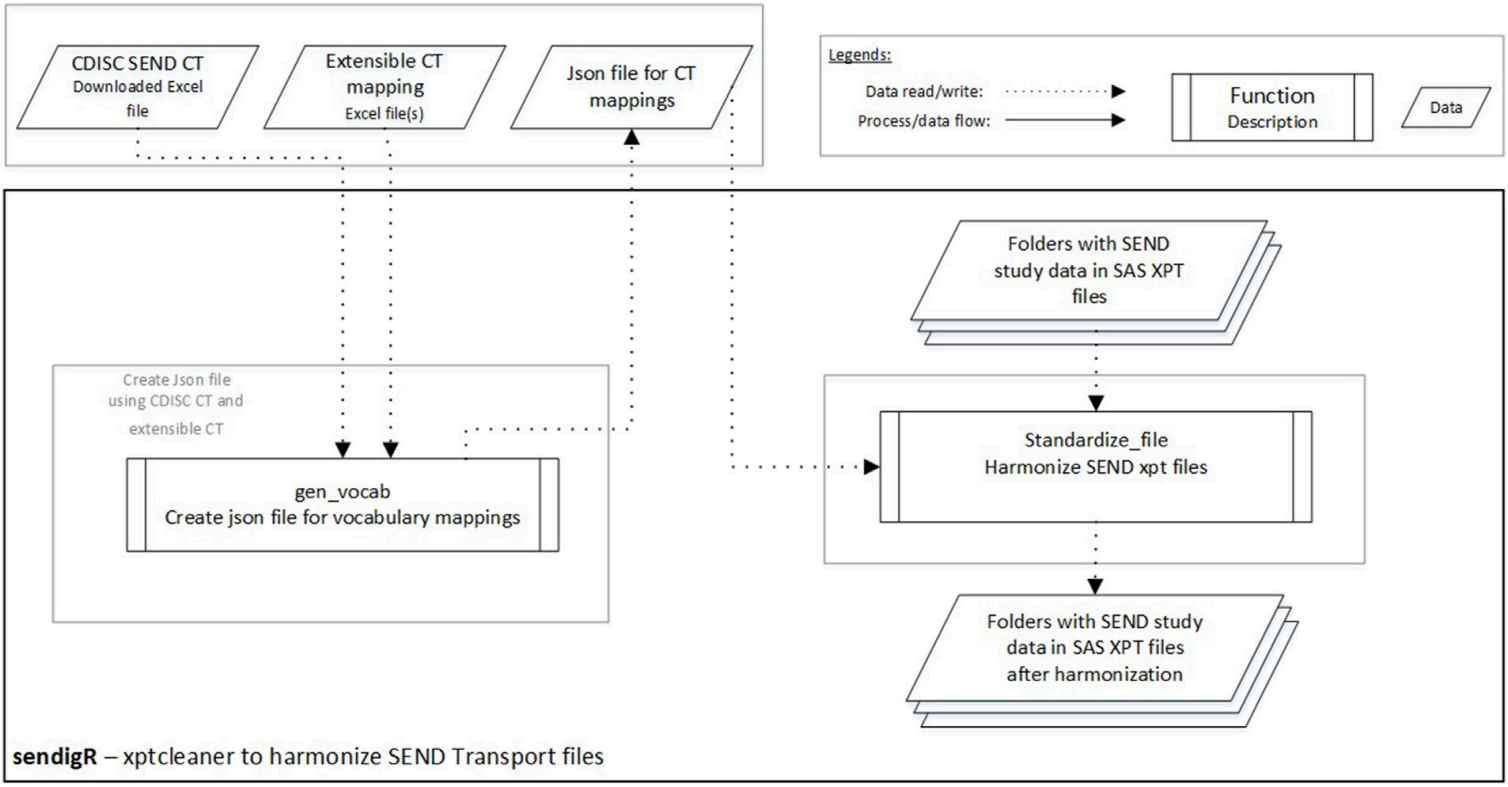
The flow diagram depicts how the xptcleaner Python module can be used to harmonize terminology in SEND XPT files.

A python module, xptcleaner, is also included in the sendigR R package and can be used to harmonize the terminology used across SEND XPT files (Figure 4). There are two main functions. A list of tab-delimited files with synonyms and preferred terms should be provided as input in the gen_vocab function, which creates a JSON file storing terminology mappings. The JSON file is then passed to the standardize_file function to harmonize the terms used in the SEND dataset XPT files. The folder containing XPT files to be cleaned is provided as input in the standardize_file function, and this function saves cleaned XPT files in the specified folder.

## Results

The value of the sendigR analytic approaches can be seen in the following examples of use cases. The first use case demonstrates how the sendigR R package can be used to help determine whether a toxicological finding of concern may be more or less likely to be treatment-related by calculating the background incidence rate of a toxicological finding of concern in an appropriately matched population of control animals, e.g., same age, species/strain, and route of administration. The second use case demonstrates how the xptcleaner Python utility (included with sendigR) can be used to ensure the accuracy of background incidence rates calculated from a sendigR-generated database by mapping synonymous terms used in SEND datasets to describe toxicological findings to appropriate controlled terminology.

**FIGURE 5:**
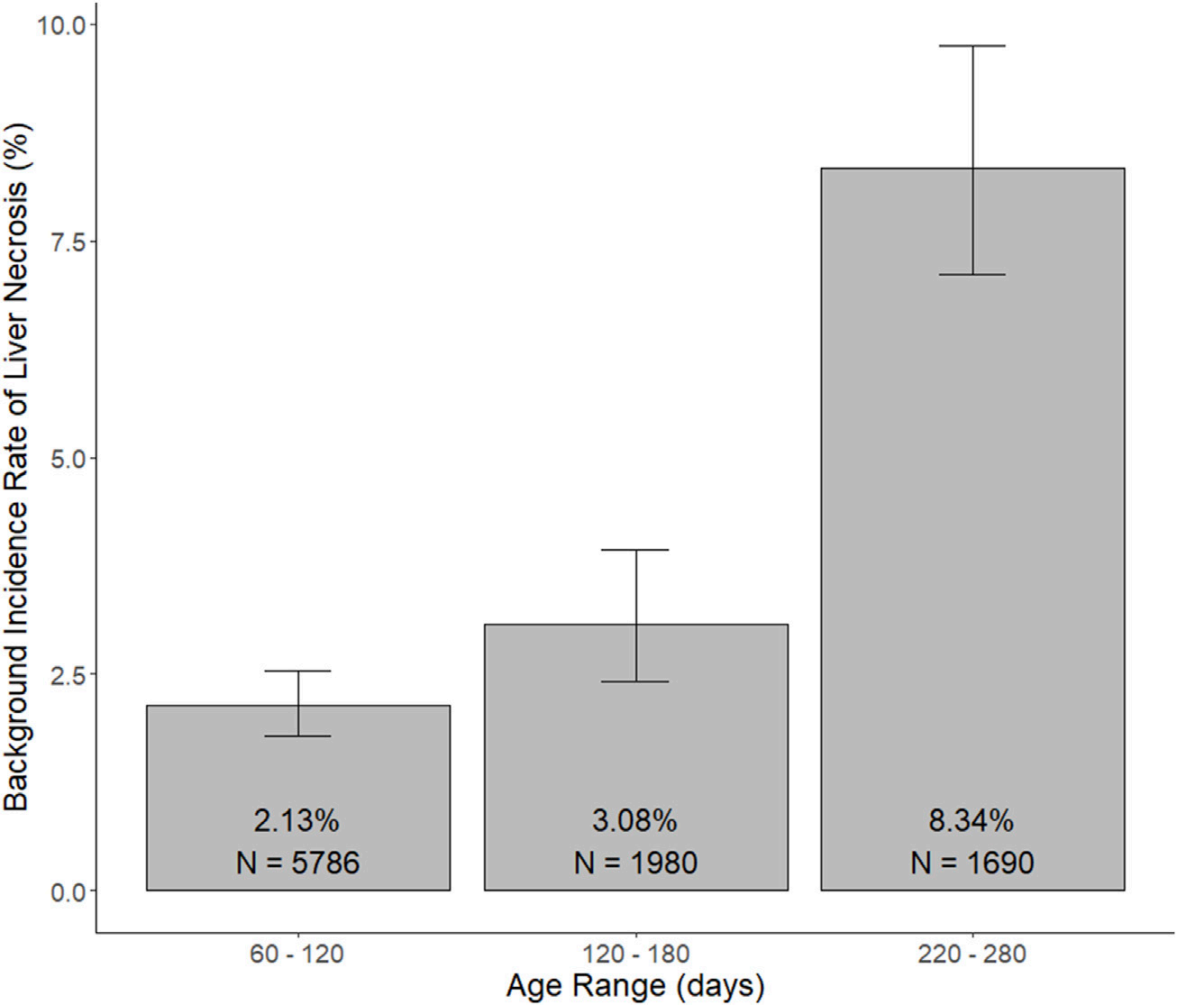
Background incidence rate of liver necrosis observed in male control rats matching the query criteria for typical age ranges at terminal sacrifice for 1-month, 3-month, and 6-month studies. The selected age ranges, i.e., 60–120 days, 120–180 days, and 220–280 days, are representative of the age ranges at terminal sacrifice for rats in studies matching the query criteria for 1-month, 3-month, and 6-month studies, respectively, such that more than 97.5% of rats from each study duration were euthanized within these respective age ranges.

Use case #1: We suppose a team of toxicologists are evaluating the safety of a new potential human therapeutic via oral gavage in repeat-dose toxicology studies conducted in rats, and they note that the incidence of a histopathologically identified finding of concern, e.g., liver necrosis, appears to be potentially dose-dependently increased for drug-treated male rats compared to control animals (control = 0/10, LD = 0/10, MD = 0/10, and HD = 1/10) in a 1-month repeat-dose toxicology study. As the treatment-relatedness of this result is quite ambiguous, consideration of the background incidence rate of the finding will be helpful. The sendigR package can be used to determine the background incidence rate at which this lesion has been observed in control animals matching the typical age ranges at terminal sacrifice for rats in 1-month repeat-dose toxicology studies. A query of a database constructed from SEND data submitted to CDER using these criteria returned a background incidence rate of 2% for this finding (Figure 5). The probability of observing this pattern of result by chance given the background incidence rate for the finding is low, suggesting that the observed effect is likely treatment-related.

Background incidence rates were also returned for queries of control animals matching the search criteria for typical age ranges at terminal sacrifice for 3-month and 6-month studies (Figure 5). The background incidence rate of liver necrosis was found to be higher, approximately 8%, in older rats at ages matching terminal sacrifice during 6-month studies. As such, the probability of observing this pattern of result would be much greater in a 6-month study, such that it would not be possible to conclude with reasonable certainty that the finding was definitively treatment-related.

Use case #2: The Python package xptcleaner enables harmonization of a SEND dataset before importing to the sendigR database. SEND data may need harmonization due to inconsistent application of standards across CROs, differences in the versions of standards being applied, or differences in extensible terminology application that make cross-study analysis difficult. xptcleaner currently supports the following controlled terminology lists: sex, strain/substrain, species, SEND severity, route of administration response, standardized disposition term, specimen, anatomical location, non-neoplastic finding type, and SEND control type. Work is underway to enhance xptcleaner to support all SEND IG-supported controlled terminology lists. Furthermore, experienced Python programmers will be able to contribute to the xptcleaner project (xptcleaner PyPI) to allow more variables to be harmonized.

**FIGURE 6:**
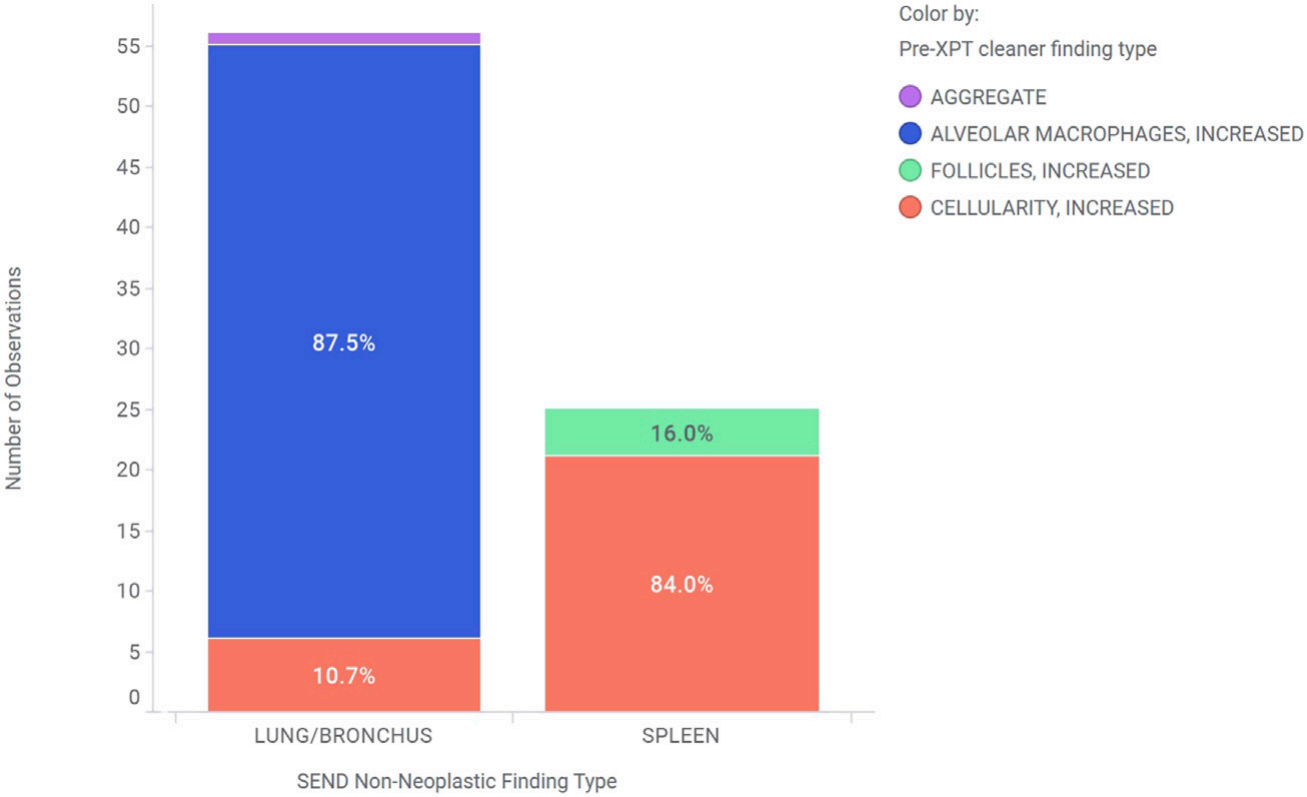
This chart shows the need for a tool like xptcleaner by showing examples of findings in the lung/bronchus and spleen tissues of control animals that were harmonized to CELLULARITY, INCREASED by xptcleaner. The bar heights represent the total number of these observations in each tissue, while the colors indicate their pre-xptcleaner non-neoplastic finding type.

Figure 6 shows an example where xptcleaner was used to clean a 2-year period of control animal data that mapped to CELLULARITY, INCREASED for the SEND non-neoplastic finding type in lung/bronchus and in spleen. While a preferred term of ALVEOLAR MACROPHAGES, INCREASED is in the current CT, this was mapped using extensible terminology to CELLULARITY, INCREASED for a statistical analysis of CELLULARITY, INCREASED across cell types. Additional extensible terms of AGGREGATE and FOLLICLES, INCREASED were mapped to CELLULARITY, INCREASED using the xptcleaner extensible term mapping feature. Therefore, prior to xptcleaner use, the number of observations of CELLULARITY, INCREASED was 6 and 21 for lung/bronchus and spleen out of 873 total animals, respectively. Applying xptcleaner with extensible terminology resulted in 56 and 24 CELLULARITY, INCREASED observations in lung/bronchus and spleen, respectively. Therefore, the prevalence of CELLULARITY, INCREASED appears to be lower in lung/bronchus (0.69%) than in spleen (2.41%) prior to xptcleaner, but is higher in lung/bronchus (6.41%) than in spleen (2.75%) after applying controlled and extensible terminology with xptcleaner. This use case highlights the value of xptcleaner to ensure that accurate and consistent controlled terminology is applied to generate reliable statistics for SEND endpoints. Furthermore, the flexibility of extensible terminology integration provides flexibility in the controlled terminology for individual use cases.

## Discussion

Currently, the functionality of the package is primarily focused on facilitating targeted extraction of historical control data based on user-specified study and/or animal parameters, e.g., date of study, route of administration of test article, species, and animal age. Additional functionality may be added to allow users to compare and contrast toxicological profiles of various test articles across studies.

• Understand the toxicity profile of a single compound across all studies performed (Ali and Snyder, 2023).
• Evaluating on- versus off-target toxicity for multiple compounds intended for the same pharmacological target (Carfagna et al., 2024).

End users who are not familiar with the R programming language are able to utilize the R Shiny web application to perform cross-study analysis. Experienced R programmers will be able to integrate the package functions into their own custom scripts/packages and potentially contribute improvements to its functionality. Programmers wishing to contribute to this development are welcome to contact us and join this free, open-source collaboration at https://github.com/phuse-org/sendigR.

## Data Availability

The datasets analyzed for this article were confidentially submitted to the FDA as part of investigational drug applications and cannot be publicly released. Requests to access these datasets should be directed to KS, kevin.snyder@fda.hhs.gov.

## Supplementary material

SUPPLEMENTARY FIGURE S1
VScreenshot of the sidebar menu used to filter historical control data by study start date, study design, route of administration, species, strain, and sex.

SUPPLEMENTARY FIGURE S2
Metadata for each selected control animal can be browsed and filtered under the ANIMALS tab.

SUPPLEMENTARY FIGURE S3
The raw SEND data records for every microscopic finding can be browsed and filtered within the MI tab under the Individual Records tab.

SUPPLEMENTARY FIGURE S4
The aggregated background incidence rates of microscopic findings can be browsed and filtered within the MI tab under the Aggregate Table tab.

SUPPLEMENTARY FIGURE S5
The aggregated reference ranges of laboratory test results can be browsed and filtered within the LB tab under the Aggregate Table tab.
