# Supplementary figures and images for: sendigR: an R package to leverage the value of CDISC SEND datasets for cross-study analysis
Source: Front Toxicol. 2024 Jul 15;6:1392686. doi: 10.3389/ftox.2024.1392686 (PMC11284615; doi:10.3389/ftox.2024.1392686)

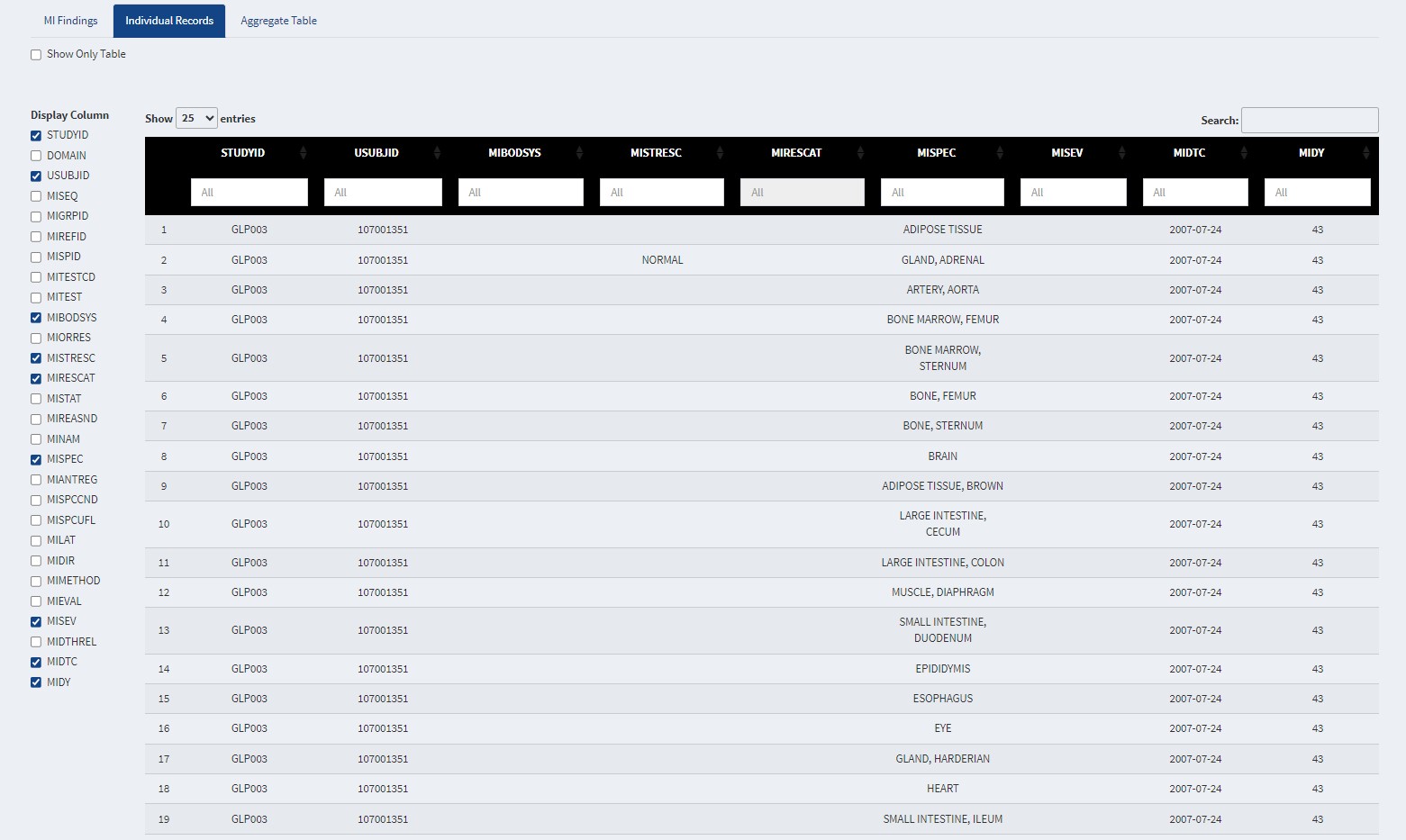

Supplement: Supplementary file 1 [file Image3.jpeg]

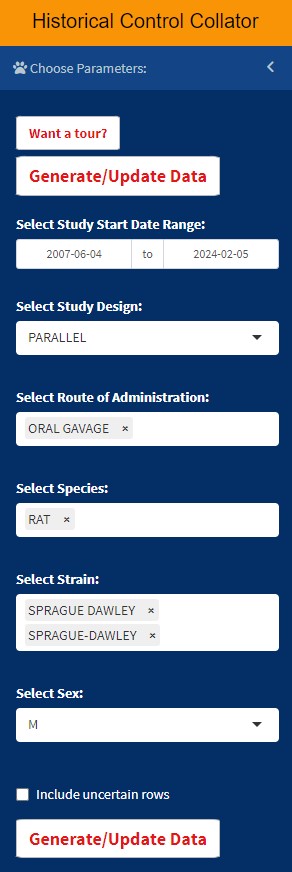

Supplement: Supplementary file 3 [file Image1.jpeg]

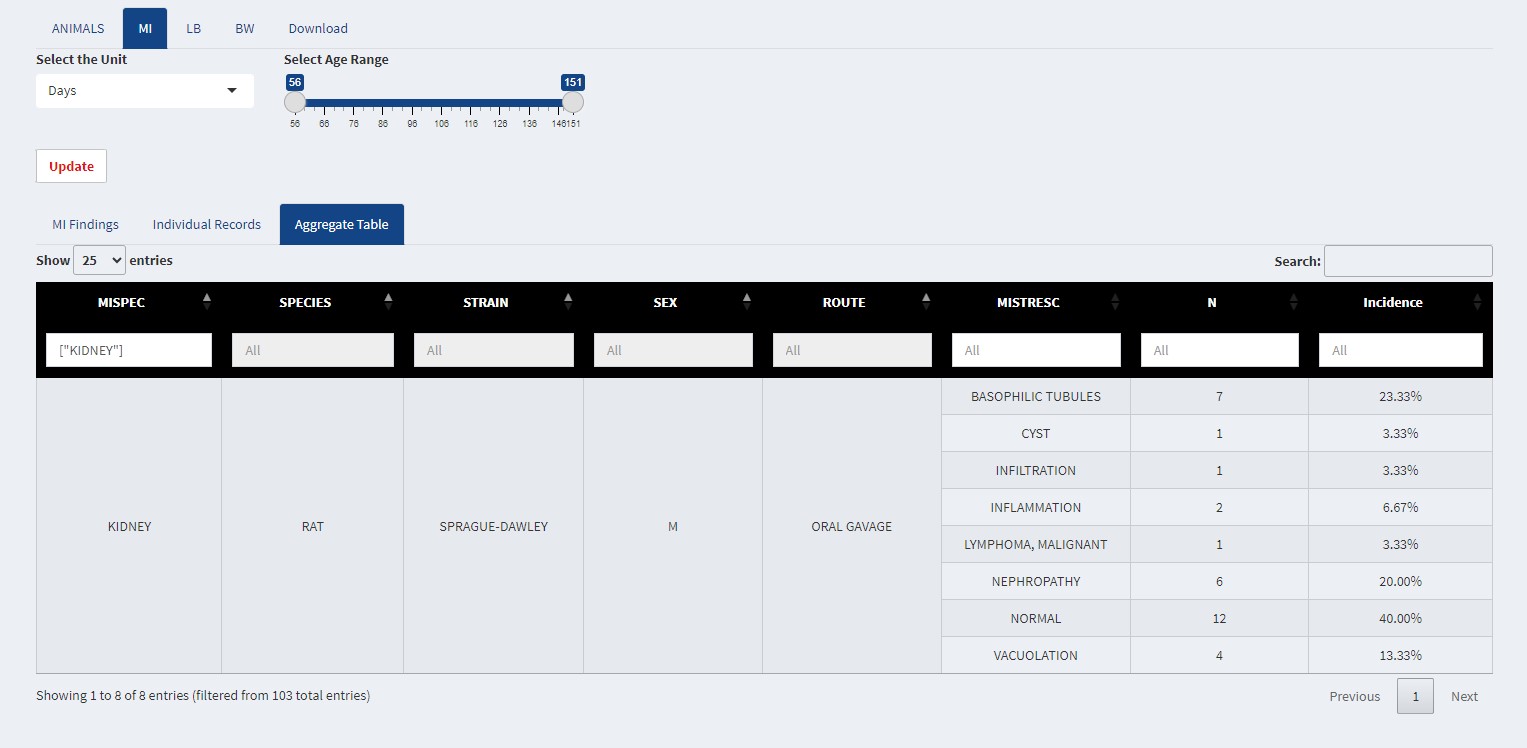

Supplement: Supplementary file 4 [file Image4.jpeg]

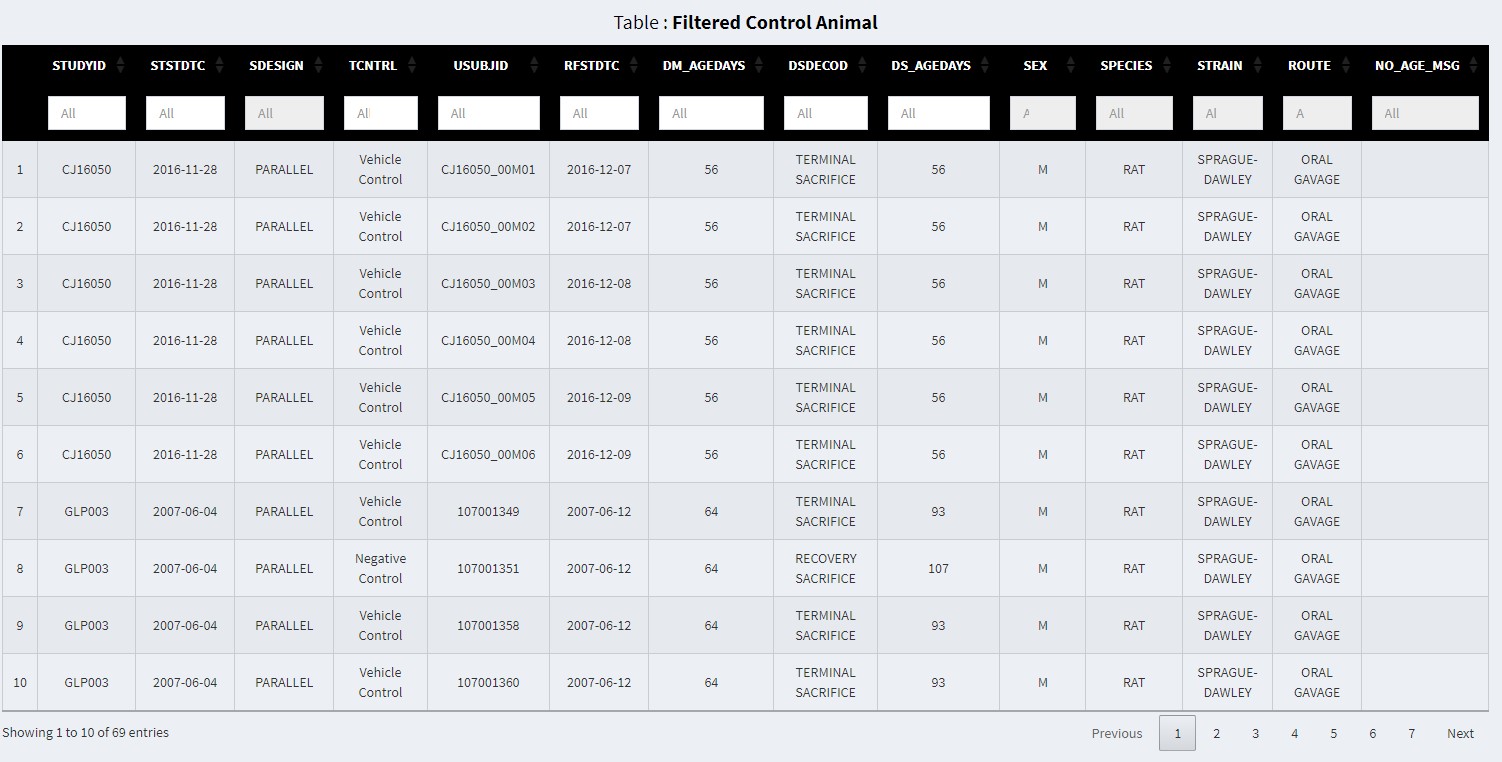

Supplement: Supplementary file 5 [file Image2.jpeg]

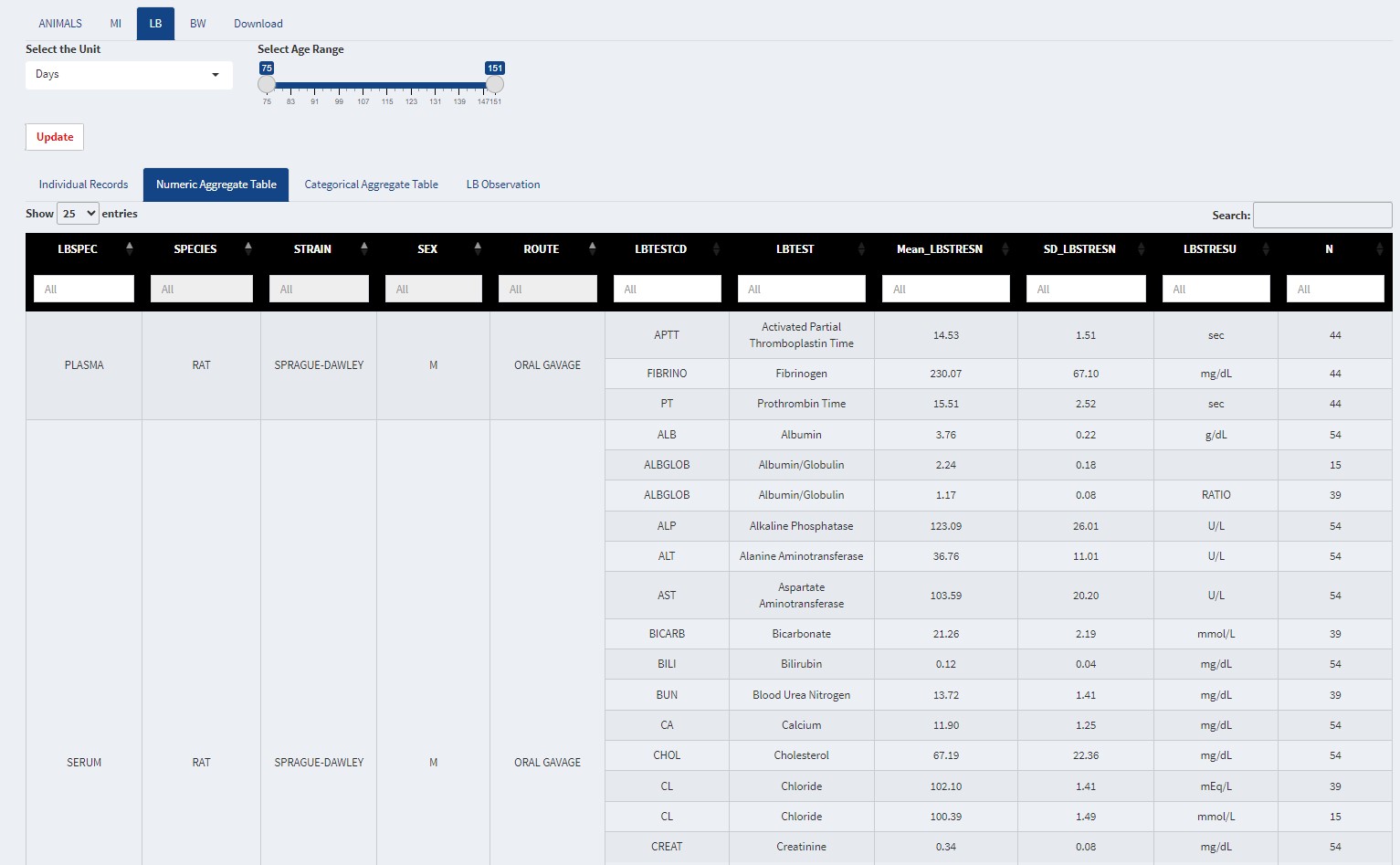

Supplement: Supplementary file 6 [file Image5.jpeg]
