# Supplementary material for: sendigR: an R package to leverage the value of CDISC SEND datasets for cross-study analysis
Source: Front Toxicol. 2024 Jul 15;6:1392686. doi: 10.3389/ftox.2024.1392686 (PMC11284615; doi:10.3389/ftox.2024.1392686)
Supplement: Supplementary file 2 [file Table1.docx]

Supplementary Material

**sendigR: an R package to leverage the value of CDSIC SEND datasets for cross-study analysis**

Kevin Snyder*, CMS Ahmed^1,2^, M Ali^1,2^, S Butler^1,2^, M DeNieu^3^, W Houser^4^, B Paisley ^5^, M Rosentreter^6^, W Wang^4^, B Larsen^7^

*** Correspondence:** Corresponding Author: Kevin Snyder* Kevin.Snyder@fda.hhs.gov

**SUPPLEMENTARY FIGURE**
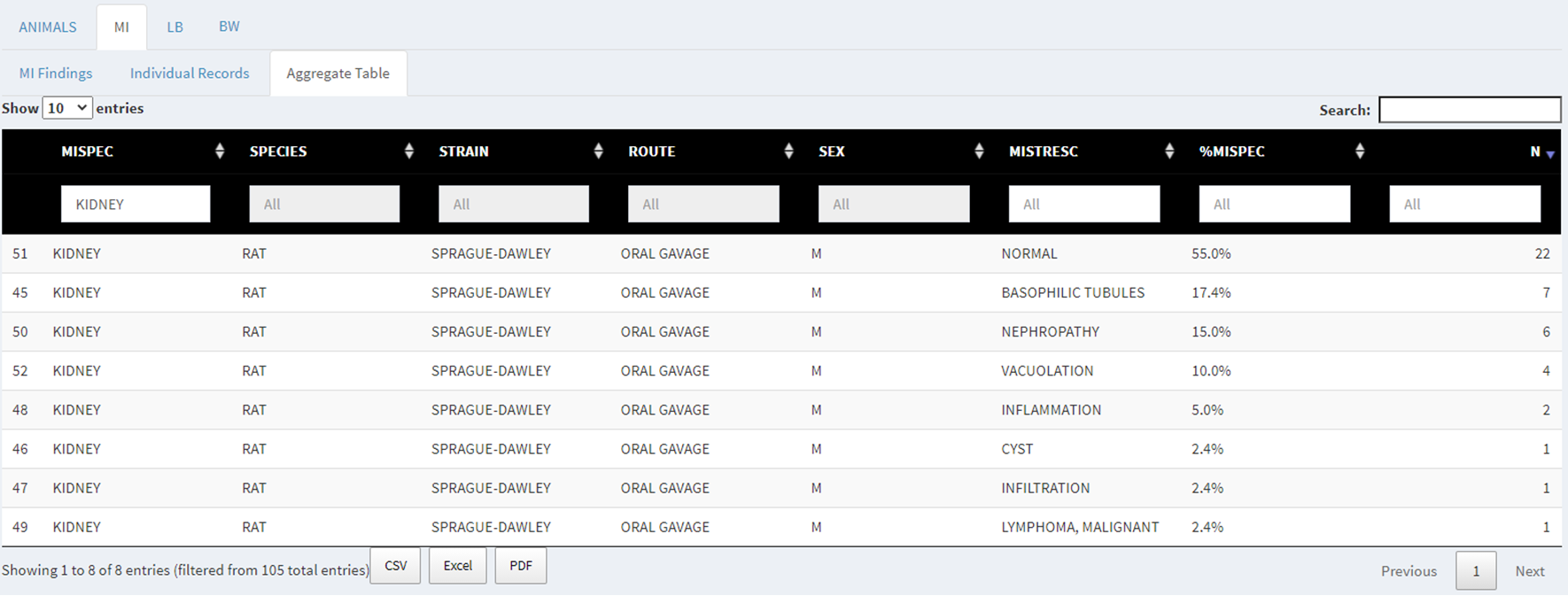
 **LEGENDS**

**B**

**C**

Supplementary Figure 1. Screenshot of the sidebar menu used to filter historical control data by study start date, study design, route of administration, species, strain, and sex.

Supplementary Figure 2. Metadata for each selected control animal can be browsed and filtered under the ANIMALS tab.

Supplementary Figure 3. The raw SEND data records for every microscopic finding can be browsed and filtered within the MI tab under the Individual Records tab.

Supplementary Figure 4. The aggregated background incidence rates of microscopic findings can be browsed and filtered within the MI tab under the Aggregate Table tab.

Supplementary Figure 5. The aggregated reference ranges of laboratory test results can be browsed and filtered within the LB tab under the Aggregate Table tab.
